# Single-cell genomics

**DOI:** 10.1093/bfgp/ely025

**Published:** 2018-07-27

**Authors:** Martin Hemberg

**Affiliations:** Wellcome Sanger Institute, Wellcome Genome Campus, Hinxton, UK

The continual drop in sequencing costs and the steady improvement of platforms and protocols have allowed single-cell sequencing to become widely used over the past few years. The ability to profile individual cells—the fundamental building blocks of all organisms—has already provided important biological insights. Crucially, the rapid development of single-cell technologies shows no sign of abating, and in the coming years, researchers will most likely present even more powerful methods that will allow for even more accurate and precise profiling of cells.

The reviews presented in this special edition of *Briefings for Functional Genomics* provide a useful guide to researchers who wish to learn more about these transformative technologies and their applications. The exciting collection clearly demonstrates the wide impact of single-cell technologies on biomedical research. The topics cover a broad spectrum, ranging from the technological platforms, via protocols and experimental design to computational analysis and applications for both basic science and the clinic.

Kolodziejczyk and Lönnberg provide an overview of the two most widely used methods for measuring the transcriptome, RT-qPCR and high-throughput sequencing. Although the latter has received the most attention, RT-qPCR remains an important tool, and the review guides the reader through the different steps of the experimental procedures. Both experimental platforms and computational analyses are covered by Ziegenhain *et al*. The review covers the relative strengths of the commonly used protocols, and it also provides an overview of the most widely used computational analysis steps.

In addition to the choices of what technologies to use when preparing the sample, an important aspect is the conceptual design of the experiment. Baran-Gale *et al*. highlight considerations that are of central importance for sound experimental design and how they apply to single-cell technologies. In particular, the article highlights the relative merits of the two most popular platforms for single-cell RNA-seq. Perhaps the most central question of experimental design is to determine how to get the most information given a finite amount of money. For single-cell sequencing experiments, this often amounts to determining the optimal trade-off between number of cells and number of reads per cell. In an elegant study, Menon investigates how three popular unsupervised clustering methods perform as the number of cells and the sequencing depth varies.

One area where single-cell RNA-seq holds great promise is for the characterization of transcriptional regulatory networks. Network analysis has been challenging using bulk methods, but with single-cell resolution the problem should, in principle, be more tractable, as the confounder of a heterogeneous population of cells can be eliminated. Fiers *et al*. provide an overview not only of the methods available for single-cell RNA-seq but also of how single-cell technologies for profiling methylation and open chromatin can be used to infer regulatory relationships.

Moving from experimental platforms and computational analysis to applications, Vegh and Haniffa present an overview of how single-cell technologies have been used in immunology. Because of the importance of rare cell types in the immune system, immunologists have been at the forefront in adapting these methods, and there have already been several landmark studies that overturned established models.

Single-cell technologies allow for very high sensitivity, and the ability to detect rare events has already been put to clinical use. Kroneis *et al*. discuss how single-cell genome sequencing can be applied to detecting microchimerisms, i.e. the presence of a small number of cells that are genetically distinct from the host. The most widespread application is for noninvasive prenatal diagnosis, but microchimerisms also feature in autoimmune diseases and cancer. Cancer can be characterized as a disease caused by damage to the genome. Thus, oncology has benefited greatly from high-throughput sequencing, as it made it possible to characterize the mutational landscape of tumors. Importantly, sequencing technology has already been put to clinical use to help identify the most promising treatments. Single-cell technologies are likely to accelerate our understanding of tumor heterogeneity, and Rantalainen argues that they will make it into clinical practice.

One of the most exciting and ambitious scientific endeavors of recent years—the Human Cell Atlas was launched in October 2016. The Human Cell Atlas is an international collaboration with the ambitious goal ‘To create comprehensive reference maps of all human cells—the fundamental unit of life—as a basis for both understanding human health, and diagnosing, monitoring and treating disease’ (humancellatlas.org). There are numerous challenges, including sample acquisition, cell handling and data processing that need to be overcome. These challenges are outlined by Hon *et al*. in an article that provides a fascinating insight into how single-cell technologies are on the verge of transforming much of biomedical research.

Although there is already an extensive literature on single-cell technologies and their applications, it is clear that the field is still in its infancy. The technologies themselves are still evolving, and scientists are busy figuring out how they can best be applied to a wide range of research questions. Thus, we can expect to see many more studies using single-cell technologies in the coming years, and the reviews in this special edition provide a guide to how this can be done.

